# Comparison of the association between measures of estrogen exposure and dementia risk in spontaneously postmenopausal women

**DOI:** 10.1002/alz70860_107743

**Published:** 2025-12-23

**Authors:** Ursula G Saelzler, Erin E. Sundermann, Margaret Gatz, Ida K Karlsson, Matthew S. Panizzon

**Affiliations:** ^1^ University of California, San Diego, La Jolla, CA, USA; ^2^ University of Southern California, Los Angeles, CA, USA; ^3^ Jönköping University, Jönköping, Sweden; ^4^ University of California San Diego, La Jolla, CA, USA

## Abstract

**Background:**

Although women account for a greater portion of Alzheimer's Disease (AD) cases than men, the mechanisms driving this sex difference remain unclear. Given its documented neuroprotective effects, estrogen exposure (or the lack thereof) has been proposed as a moderator of AD risk in women. Specifically, greater estrogen exposure during the premenopausal period is hypothesized to provide prolonged neuroprotection through older adulthood, thereby reducing AD risk, while an earlier transition to the low‐estrogen postmenopausal state may increase AD risk.

**Method:**

To examine these hypotheses, we fit a series of Cox proportional hazards models to data from 9,793 spontaneously postmenopausal women in the Swedish Twin Registry. Chronological age was used as the model time scale and age 65 was designated as the start of model time. Women whose data collection occurred after age 65 were included as delayed entrants to prevent survivor bias in the results. Over an average of 14.43 years of follow‐up 1,241 women developed dementia.

**Result:**

Covarying for smoking history, education, and number of births, both reproductive span and age of spontaneous menopause showed significant non‐linear relationships with dementia risk, where greater deviations from the mean were associated with increased risk. The models exhibited identical concordance values (0.57, SE = 0.009), indicating that both measures explain only a small proportion of the variance in dementia onset and that the models do not differ in their ability to correctly predict the order of dementia onset.

**Conclusion:**

These results suggest that estrogen exposure plays a role in individual differences in dementia risk. However, the comparable concordance values across models limit the ability to further probe mechanistic explanations. Notably, although reproductive span is a commonly used index of lifetime estrogen exposure, it is inherently highly correlated with age of menopause given the larger variance in menopause age (here 15.66) compared with age of menarche (here 2.38). Future work employing more comprehensive measure of lifetime estrogen exposure are warranted to explore the mechanisms underlying the estrogen‐AD connection.

